# Phylogeographic and Demographic Analysis of the Asian Black Bear (*Ursus thibetanus*) Based on Mitochondrial DNA

**DOI:** 10.1371/journal.pone.0136398

**Published:** 2015-09-25

**Authors:** Jiaqi Wu, Naoki Kohno, Shuhei Mano, Yukio Fukumoto, Hideyuki Tanabe, Masami Hasegawa, Takahiro Yonezawa

**Affiliations:** 1 School of Life Sciences, Fudan University, SongHu Rd. 2005, Shanghai, 200438, China; 2 Department of Geology and Paleontology, National Museum of Nature and Science, Amakubo 4-1-1, Tsukuba, Ibaraki, 305–0005, Japan; 3 Graduate School of Life and Environmental Sciences, University of Tsukuba, Tennoudai 1-1-1, Tsukuba, Ibaraki, 305–8572, Japan; 4 The Institute of Statistical Mathematics, Midori-cho 10–3, Tachikawa, Tokyo, 190–8562, Japan; 5 Hiroshima City Asa Zoological Park, Asakita-ku, Hiroshima city, Hiroshima, 731–3355, Japan; 6 School of Advanced Sciences, The Graduate University for Advanced Studies, Shonan Village, Hayama, Kanagawa, 240–0193, Japan; University of York, UNITED KINGDOM

## Abstract

The Asian black bear *Ursus thibetanus* is widely distributed in Asia and is adapted to broad-leaved deciduous forests, playing an important ecological role in the natural environment. Several subspecies of *U*. *thibetanus* have been recognized, one of which, the Japanese black bear, is distributed in the Japanese archipelago. Recent molecular phylogeographic studies clarified that this subspecies is genetically distantly related to continental subspecies, suggesting an earlier origin. However, the evolutionary relationship between the Japanese and continental subspecies remained unclear. To understand the evolution of the Asian black bear in relation to geological events such as climatic and transgression-regression cycles, a reliable time estimation is also essential. To address these issues, we determined and analyzed the mt-genome of the Japanese subspecies. This indicates that the Japanese subspecies initially diverged from other Asian black bears in around 1.46Ma. The Northern continental population (northeast China, Russia, Korean peninsula) subsequently evolved, relatively recently, from the Southern continental population (southern China and Southeast Asia). While the Japanese black bear has an early origin, the tMRCAs and the dynamics of population sizes suggest that it dispersed relatively recently in the main Japanese islands: during the late Middle and Late Pleistocene, probably during or soon after the extinction of the brown bear in Honshu in the same period. Our estimation that the population size of the Japanese subspecies increased rapidly during the Late Pleistocene is the first evidential signal of a niche exchange between brown bears and black bears in the Japanese main islands.

This interpretation seems plausible but was not corroborated by paleontological evidence that fossil record of the Japanese subspecies limited after the Late Pleistocene. We also report here a new fossil record of the oldest Japanese black bear from the Middle Pleistocene, and it supports our new evolutionary hypothesis of the Japanese black bear.

## Introduction

The Asian black bear (*Ursus thibetanus*) is a middle-sized bear that is widely distributed in Asia, from Japan in the east to Iran in the west. Fossil evidence suggests a broader distribution area in the past, that extended to Southern Siberia (near Lake Baikal), East Europe (Moldova) and West Europe (Germany and France) [1–3].

While *U*. *thibetanus* is adapted to broad-leaved deciduous forests [1,4], it is omnivorous, and the Japanese population, in particular, depends more on vegetation than meat [1]. On the other hand, these bears are more aggressive than the American black bear (*U*. *americanus*) and the Old World brown bear (*U*. *arctos*), and they sometimes kill animals such as cattle, sheep, ponies and occasionally humans [4]. In spite of their ecological importance, little is known about the evolutionary history of *U*. *thibetanus*.

*U*. *thibetanus* is classified into several subspecies [2,5,6] and, according to Steinmetz and Garshelis [7], can be divided into three extant populations. The first population occupies all continental Southeast Asian countries except Malaysia, and is also found in patches in southern China. This population also has narrow latitudinal distribution from southeastern Iran eastward through Central Asia (Pakistan and Afghanistan), and the foothills of the Himalayas. The second population is distributed in northeastern China, the southern Russian Far East, and into North Korea, as well as South Korea. The third population is found in the Japanese Islands including Honshu, Shikoku and Kyushu, but the local population in Kyushu has probably gone extinct [8]. We refer to these populations as the south continental population, the north continental population and the Japanese population, respectively.

Recent molecular phylogeographic analyses have clarified that the Japanese population is genetically distant from the two continental populations, and suggested its earlier origin [8,9]. Subsequently, to evaluate the genetic diversity of the continental population, Kim et al. [10] extensively collected samples both from the north and the south continental population (the latter from Vietnam) and reconstructed a phylogenetic tree that included the Japanese population. Their resulting tree indicated that the north continental population and the Japanese population evolved relatively recently from the south continental population.

Despite these studies, evolutionary time estimates for Asian black bear populations are still controversial. For example, the divergence time between the Japanese and continental population (both north and south) has been estimated as around 0.5 Ma (Mega annum) [9] and 1.4–2.6 Ma [8]. Both studies used divergence times at the genus (*Ursus*) level as calibration points: Yasukochi et al. [9] assumed that the split between *U*. *arctos* and *U*. *spelaeus* was 1.2 Ma based on the estimate in a previous study [11] using D-loop sequences, while Ohnishi et al. [8] assumed that the split between *U*. *thibetanus* and *U*. *americanus* occurred at 2.0 to 3.5 Ma, based on fossil records [12]. However, as will be discussed later, the suitability of these calibrations needs to be carefully examined.

To understand the evolution of the Asian black bear in relation to geological events such as climatic and sea-level changes, with transgression-regression cycles, reliable dating is essential. To address this issue, we determined and analyzed the mitochondrial genome sequence of an Asian black bear from the Japanese population (Japanese black bear; *Ursus thibetanus japonicus*). In previous studies, divergence times were estimated from *cytochrome b* [9] or D-loop sequences [8]. However, time estimation on the basis of D-loop sequence is often difficult because of the underestimation of internal branch lengths (particularly of those near the root) caused by multiple substitutions at the same sites, especially in hyper-variable regions [13]. In addition, longer sequences provide a smaller standard error [14] and a more robust estimate [15].

It is important to note that divergence times within the family Ursidae are still controversial. For example, two studies [16,17] estimated divergence times on the assumption that the giant panda (*Ailuropoda melanoreuca*) separated from the other bears around 12 Ma, based on fossil evidence [18,19]. The estimates of divergence times among the genus *Ursus* were from the Late Pliocene to the Pleistocene (2~3 Ma) [16, 17]. On the other hand, Arnason et al. [20] estimated the divergence time between *A*. *melanoreuca* and the other ursids to be 30 Ma, and the divergence times of the genus *Ursus* to be in the Late Miocene (6~8 Ma). These discrepancies must come from a different choice of fossil records for the divergence time estimates, and depend on the accuracy of the geological ages of such fossil records. Thus, a more reliable estimation of divergence times among the Ursidae is needed. In this study, we first estimate the divergence times of all Ursidae species, in the framework of the whole Carnivoran evolution, with reliable fossil calibrations and superior molecular evolutionary models. We then estimate the coalescent times among *U*. *thibetanus* as well as the behavior of the population size over time, through its history on a geological timescale.

## Materials and Methods

### 2.1 Ethics statement

All of the experimental work involving animals in this study followed the guidelines of the Animal Experimental Ethics Committee of the School of Advanced Sciences, The Graduate University for Advanced Studies, Japan, and was approved by the Committee.

### 2.2 Sample and sequencing

A blood sample of Japanese black bear was provided by Asa Zoological Park (Hiroshima, Japan). This individual was captured in Hiroshima Prefecture. The sample was stored at -20°C until used. Genomic DNA was extracted by standard phenol-chloroform extraction [21]. The complete mitochondrial genome sequence was determined using the procedures described by Nikaido et al. [22]. The nucleotide sequence of the mitochondrial genome of Japanese black bear determined in this study was deposited in the DDBJ (accession number: AB863014).

### 2.3 Phylogenetic analysis and divergence time estimation among Carnivora

Complete mitochondrial genome (mt-genome) sequences of 71 species of Carnivora and one species of Pholidota were downloaded from GenBank; accession numbers are listed in S1A Table. All 12 protein-coding genes in the heavy strand of mt-genomes were aligned separately by using MUSCLE program and then concatenated. Start codons, stop codons and overlapping regions (between *ATP6* and *ATP8*, *ND4* and *ND4L*, and *ND5* and *ND6*) were removed, and the resulting total sequence length was 10,704 bp. A phylogenetic tree was inferred by RAxML 7.2.8 [23,24] using the GTR+Γ+I model [25–27]. The difference among the three codon positions was taken into account by a partition model.

After tree inference, divergence time was estimated based on the same dataset, using the MCMCtree program of PAML 4.6 [28] with the codon substitution model [29]. Since the accuracy of the branch length estimates is directly related to the divergence time estimations, the more realistic model was preferred for this analysis. Although the codon substitution model is inappropriate for a heuristic tree search due to the huge computational burden, the superiority of this model compared with standard nucleotide substitution models (e.g., GTR model) and amino acid substitution models were demonstrated by our previous studies [30, 31]. The selection of the fossil calibration method is also an important factor in divergence time estimation. Although several studies simply apply the oldest fossil record of the Ursidae as a calibration point, fossil records in general do not point to the true time of divergence because the first stratigraphic appearances of taxa in the fossil records are subject to sporadic sedimentary hiatuses due to erosion or to a lack of sedimentation during regression and/or irregular sedimentary processes. Because of these uncertainties, an assumed phylogeny implies such gaps if two sister taxa have different times of first appearance or if a gap exists between the last appearance of an inferred ancestor and the first appearance of its inferred descendant [30]. For this reason, the use of multiple reliable fossil records is preferred for time estimation. The calibration points derived from fossil records were the same as in our previous studies [30,31].

### 2.4 Phylogenetic analysis and coalescent time estimation within *U*. *thibetanus* based on the mitochondrial genome

A new dataset, including eight complete mitochondrial genomes of Asian black bears, was used in this analysis. Accession numbers are listed in S1B Table. The American black bear, brown bear and sloth bear were used as out-groups. Methods for data preparation and phylogenetic inference are the same as in Subsection 2.2 except that 12S rRNA and 16S rRNA were also involved. The GENETREE program ver. 9.0 (http://www.stats.ox.ac.uk/~griff/software.html) [32] was also used for the inference of genealogy and coalescent times. Since this method assumes neutrality, only the 3^rd^ codon positions, which are generally regarded as neutrally (or nearly neutrally) evolving sites, were used. Among 3173 3^rd^-codon position sites, 469 were variable. To make this data set compatible with the infinite site model, sites with multiple substitutions were excluded, and finally 438 sites remained. However, since the GENETREE program did not work on this data set, probably due to an excessive number of sites, we separated the data into six fragments and analyzed them one by one.

Hasegawa et al. [33] indicated a higher ω ratio (non-synonymous substitution rate/synonymous substitution rate) in an intra-species comparison than in an inter-species comparison. Ho et al. [34] demonstrated the time dependence of the evolutionary rate, and found a higher evolutionary rate in the short term (<1~2 Myr) and a lower evolutionary rate in the long term (>1~2 Myr). This is probably due to slightly deleterious mutations that were not completely swept from the populations over a short evolutionary period. The coalescent times within Asian black bear were estimated, using synonymous substitution sites that were regarded as nearly neutral [35]. By applying the estimated divergence times among Carnivora as calibration points, the coalescent times within Asian black bear were estimated using the MCMCtree program with the GTR+Γ model [25,26,28].

### 2.5 Demographic analysis of *U*. *thibetanus* based on D-loop data

We downloaded D-loop sequence data that had been determined in previous studies [8,10], and aligned them together with the D-loop sequences of the mitochondrial genome data listed in the S1B Table. It should be noted that since the D-loop sequence region of the *U*. *thibetanus mupinensis* (DQ402478) is identified as that of American black bear (data not shown), we did not use the D-loop sequence of this individual in the following analyses.

We first divided Asian black bears into two populations, namely a Japanese population and a continental population. As mentioned above, the continental population was also divided into two populations: the south continental population (Chinese and Vietnamese population) and the north Asian continental population (Russian and North Korean population) due to their geographical distribution pattern [36].

The samples from the continental population comprise 44 individuals: the south continental population (17 individuals) and the north continental population (26 individuals), and one individual of unknown locality. The ‘all Asian black bear data set’ (64 individuals) consists of 44 individuals from continental populations and 20 individuals from the Japanese population.

Furthermore, we divided the Japanese population into three sub-populations: the western sub-population, the eastern sub-population and the southern sub-population [8, 9]. The Japanese population data set consisted of 133 individuals from a western sub-population (60 individuals), an eastern sub-population (60 individuals), and a southern sub-population (13 individuals).

Phylogenetic relationships were inferred using the ML (maximum likelihood) method and the median joining (MJ) network method [37]. The ML tree was inferred using the RAxML program with the GTR+I+Г model. Since the D-loop sequences are short and possess homoplasy caused by their high substitution rate at limited hypervariable sites, tree inference based on D-loop data only is not as reliable as inference based on the mt-genome. Therefore, the binary-backbone was given a priori, as a constraint of the phylogenetic relationships, on the basis of the result of the mt-genome analysis. The MJ network was inferred using the NETWORK program ver. 4.6.11 (http://www.fluxus-engineering.com/sharenet.htm).

Since the strict molecular clock model could not be rejected by a likelihood ratio test, we carried out a new calculation of the evolutionary rate of the D-loop based on 61 Asian black bears including individuals from the continental population and Japanese population, assuming the split of *U*. *t*. *formosanus* and others (e.g., *U*.*t*. *thibetanus*, *U*.*t*. *ussuricus*) as 0.51 Ma (the estimated coalescent time in this study, based on the mt-genome data). The evolutionary rate of the D-loop was found to be 2.69×10^−8^ per site per year. Using this evolutionary rate, the ancestral population sizes of Japanese and continental populations were estimated using the BEAST program ver. 1.7.4 [38] with a Bayesian Skyline Plot method [39]. Watterson’s Θ [40], the Θ on the basis of nucleotide diversity [41], and Tajima’s D [41] were estimated by the DNASP program ver. 5.10.01 [42]. In this analysis, all sequence data used by Ohnishi et al. [8] were applied for the Japanese population.

The reliability of the demographic analysis on the basis of D-loop data seemed to be questionable because of the short sequence length and homoplasy of the D-loop. With the aim of addressing this issue, we examined the reliability of the D-loop data for the demographic analysis on the basis of the real and simulated data, and confirmed that the results from the D-loops and the complete mitochondrial protein coding genes showed essentially the same tendency (see S1 Text and S1 Fig).

## 3. Results and Discussion

### 3.1 Divergence time within the family Ursidae

**Fig 1:**
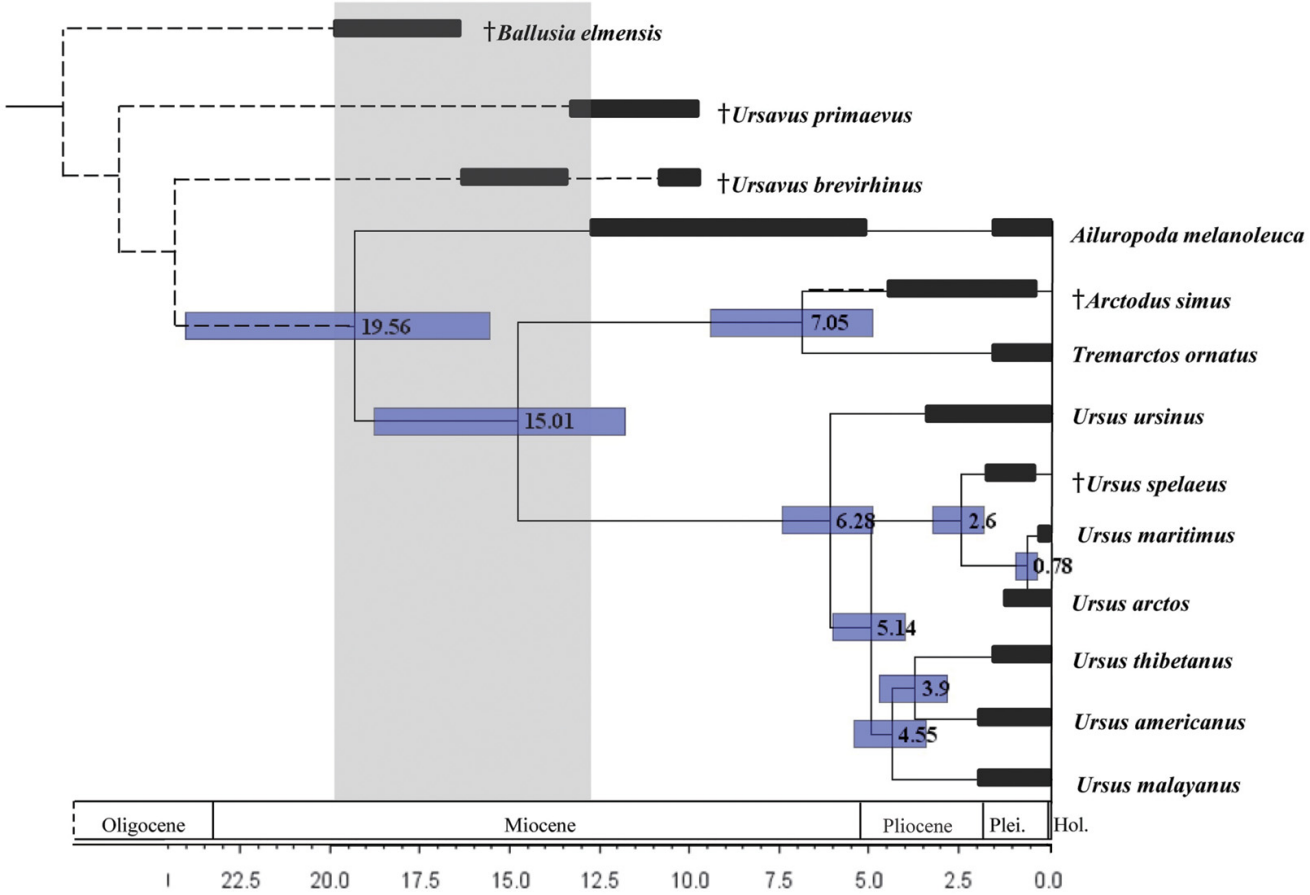
Divergence times of the family Ursidae. Nodal numbers indicate the estimated divergence time (Ma). Horizontal gray bars spanning the nodes mark the 95% confidence interval for the divergence time. The phylogenetic positions of extinct lineages, which are indicated by the dashed lines, follow [18]. Horizontal black bars indicate temporal range based on fossil evidence. The temporal range of the common ancestor of Ursidae based on fossil evidence is indicated by the shading. Extinct species are marked by a dagger (†).

A ML tree inferred from the complete mitochondrial protein genes is shown in S2 Fig. This tree topology is in general agreement with previous studies based on mitochondrial genomes (for Musteloidea [30,43], Otarioidea [31], Phocidae [20,44,45] and Ursidae [42,46]). Divergence times were estimated based on this tree topology except for the assumption of monophyly of the Musteloidea-Pinnipedia [20], and the result is shown in Fig 1 (a magnified view of the Ursidae is displayed).

The divergence time between Ursinae+Tremarctinae (bears) and Ailuropodinae (giant panda) was estimated to be 19.6±1.6 Ma. The divergence time between the Ursinae (bears other than spectacled bear) and Tremarctinae (spectacled bear) was estimated to be 15.0±1.3 Ma, and the basal split of the crown Ursinae (genus *Ursus*) was estimated to be 6.3±0.8 Ma. These estimates are much younger than those of Arnason et al. [20] and much older than those of Zhang and Ryder [16], but similar to those of Krause et al. [46].

Interestingly, although no fossil record within the Ursidae was used in the calibration of our analyses, our estimates are compatible with the known fossil records of Ursidae (Fig 1). The age of the basal split of the crown taxa should be older than the oldest known record (OKR) of the crown taxa, and younger than the OKR of the stem taxa [30,47]. The OKR of the crown Ursidae is *Kretzoiarctos beatrix* from the Middle Miocene [18] and the OKR of the stem Ursidae is *Ballusia elmensis* from the Early Miocene (MN 3, 20.5–18.0 Ma [48]). Therefore, the age of the basal split of the crown Ursidae (divergence time between Ursinae+Tremarctinae and Ailuropodinae) should be between 12.9 Ma and 20.5 Ma. Our estimate is very close to the older limit. The timing of the divergence of the genera *Tremarctos* and *Arctodus* (7.05 Ma) slightly predates the first appearance of the OKR of these genera. Our time estimates as well as the inconsistent pattern of gene tree topologies of the Ursidae [49] suggest that there were rapid speciation events within the genus *Ursus* from the Late Miocene to the Pliocene (6.3~2.6 Ma, this study). The OKR of this genus is also consistent with these timings (MN 14, 5.3–4.2 Ma, Early Pliocene, [50]). These fossil records suggest that our estimates are reliable.

Krause et al. [46] used the youngest fossil record of the genus *Ursavus*, which was assumed to be a direct ancestor of the genus *Ursus*, as the maximum age of the genus *Ursus*. However, there is still room to reconsider this calibration because the phylogenetic position of the genus *Ursavus* remains controversial. *Ursavus* is recognized as a stem taxon for the crown Ursidae, and not a direct ancestor of the genus *Ursus* [18, 51]. Also, *Ursavus* is somewhat heterogeneous, and recent studies suggest that several species within *Ursavus* should be transferred to other genera [48, 50, 52]. However, at least seven *Ursavus* species are recognized from Eurasia during the Middle to Late Miocene [52], and the age of the youngest fossil record of *Ursavus* (MN 11, 8.7–8.0 Ma, Late Miocene [50]) and time of the basal node of *Ursus* (6.3±0.8 Ma, this study) are seemingly concordant. This suggests that the extinction of *Ursavus* triggered successive speciations within the genus *Ursus*.

In addition, the timing of the successive speciations of *Ursus* (Late Miocene to Pliocene) is in agreement with the global cooling event starting from the Late Miocene [53]. This suggests that the spread of savanna and open scrubland [54], and fragmentation of the forests, resulted in reductions of gene flow among the subpopulations of the common ancestral species of *Ursus* and enhanced the rapid speciation of *Ursus*. As a result, *Ursus* species received a fundamental shift of their ecological niches.

Indeed, other Carnivoran families such as Felidae, Mustelidae and Otariidae also show successive speciation events during this period [30,31]. However, it is possible that the mechanisms that enhanced the rapid speciation are different for each taxon. In the case of Felidae and Mustelidae, environmental change may have enhanced their adaptive radiation to grassland (coevolution with rodents) and acted as a driving force of the speciation as in response to successive changes in the food web, which is relevant to the evolution of these families [30]. In the case of Otariidae, changes in their distribution areas triggered the accidental transit of the biological barrier, and resulted in the reclamation of distribution areas [31]. In the case of the Ursidae, fragmentation of forests seems to have played a role in vicariance, with the isolation of each subpopulation resulting in rapid speciation. Inconsistency of the tree topologies among individual gene loci [49] implies a large ancestral population and successive speciations.

### 3.2 Genealogy and coalescent times of Asian black bear based on the mt-genome

**Fig 2:**
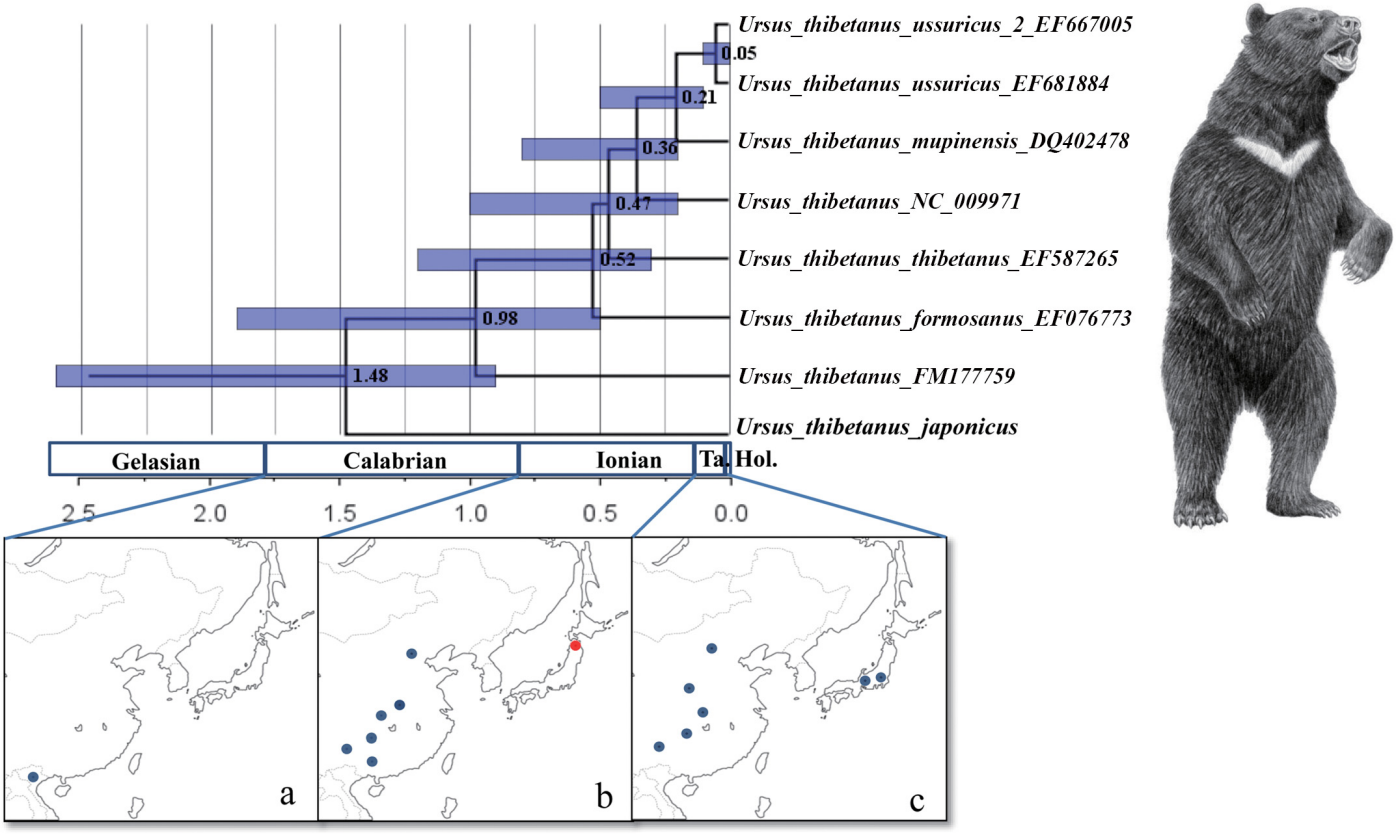
Coalescent times of the Asian black bear. Nodal numbers indicate the estimated coalescent time (Ma). Horizontal gray bars show the 95% confidence interval for the coalescent times. (a-c) Geographical distribution of fossils of Asian black bear in (a) the Calabrian (Early Pleistocene) [58], (b) the lonian (Middle Pleistocene) [58, Kohno, unpublished] and (c) the Tarantian (Late Pleistocene) [58, 60]. The illustration is of a Japanese black bear (*Ursus thibetanus japonicus*), kindly provided by Utako Kikutani. Circles on maps indicate the fossil record of Asian black bears (Blue circles indicate the fossil records reported by previous studies and red circle indicates the fossil records newly reported by this study.).

The ML tree of eight Asian black bears, as inferred from the complete mitochondrial protein genes and rRNAs, is shown in S3 Fig The time-calibrated tree is shown in Fig 2. American black bear (*U*. *americanus*), brown bear (*U*. *arctos*) and sloth bear (*U*. *ursinus*) were used as out-groups.

The Japanese sub-species (*U*. *thibetanus japonicus*) was recognized as the basal split among the species *U*. *thibetanus* in this tree, indicating that the Japanese population is not a direct descendant of the (extant) continental populations. The fossil record (e.g., the Early Pliocene fossil from Moldova [3]) suggests that the initial population of Asian black bear dispersed throughout the Eurasian continent, and it is possible that the Japanese population is a relic of such an ancient population.

**Fig 3:**
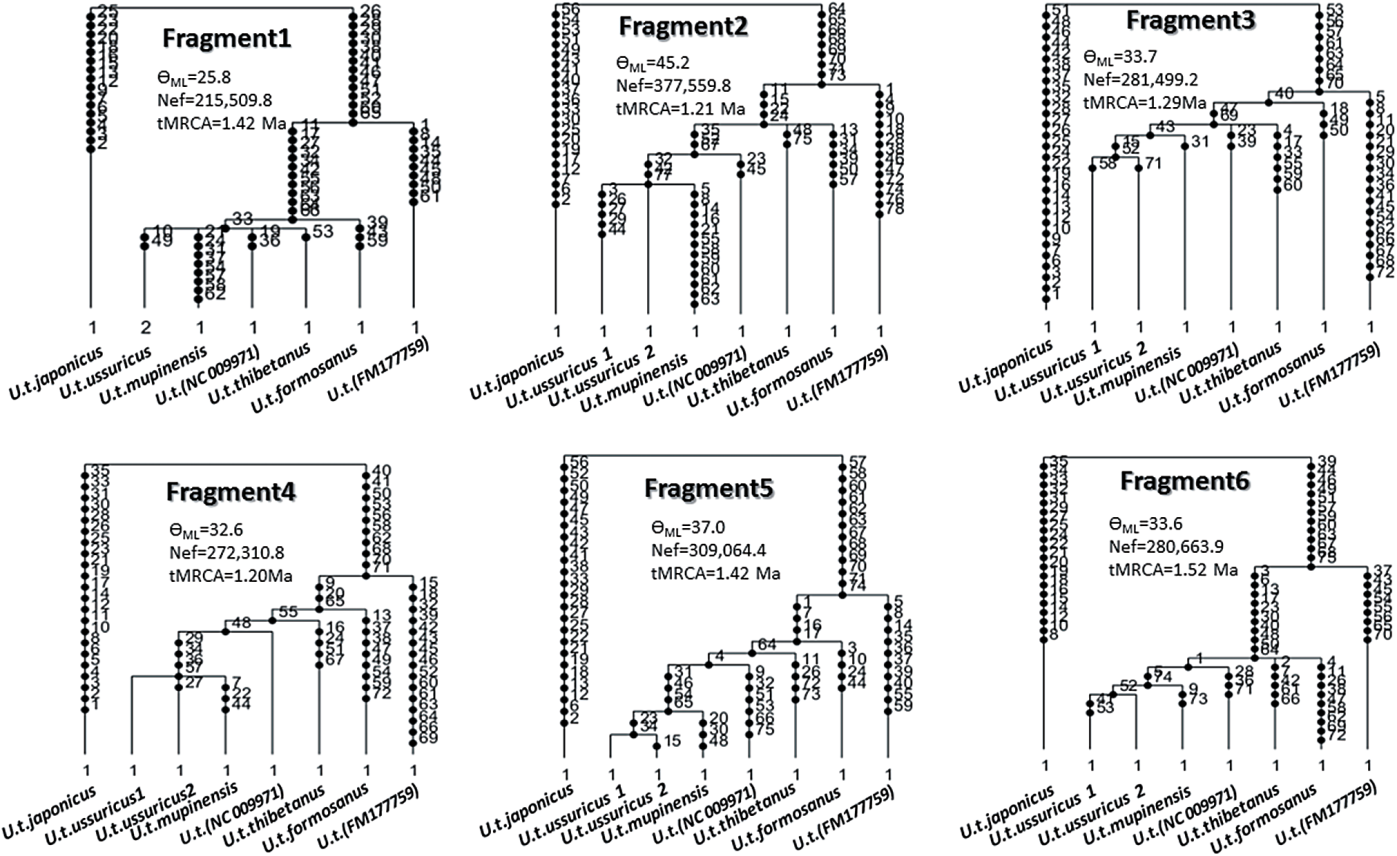
Genealogy of eight Asian black bears as inferred from the 3rd codon positions of the complete mitochondrial protein genes using the GENETREE program. Since the program did not work on the whole data set, probably due to excessive numbers of mutation sites, the whole data set was separated into six fragments. The dots on the branches indicate the number of mutation sites. The values of ρ_ML_ (= 2×Nef×μ; μ is the mutation rate per sequence per generation) and tMRCAs were also estimated by GENETREE. The substitution rate of the 3rd codon positions of mitochondrial protein genes of Asian black bear was estimated to be 3.03×10^−8^/site/year (data not shown), and the average mutation rate of each of the six fragments is 5.99×10^−5^/sequence/generation.

**Fig 4:**
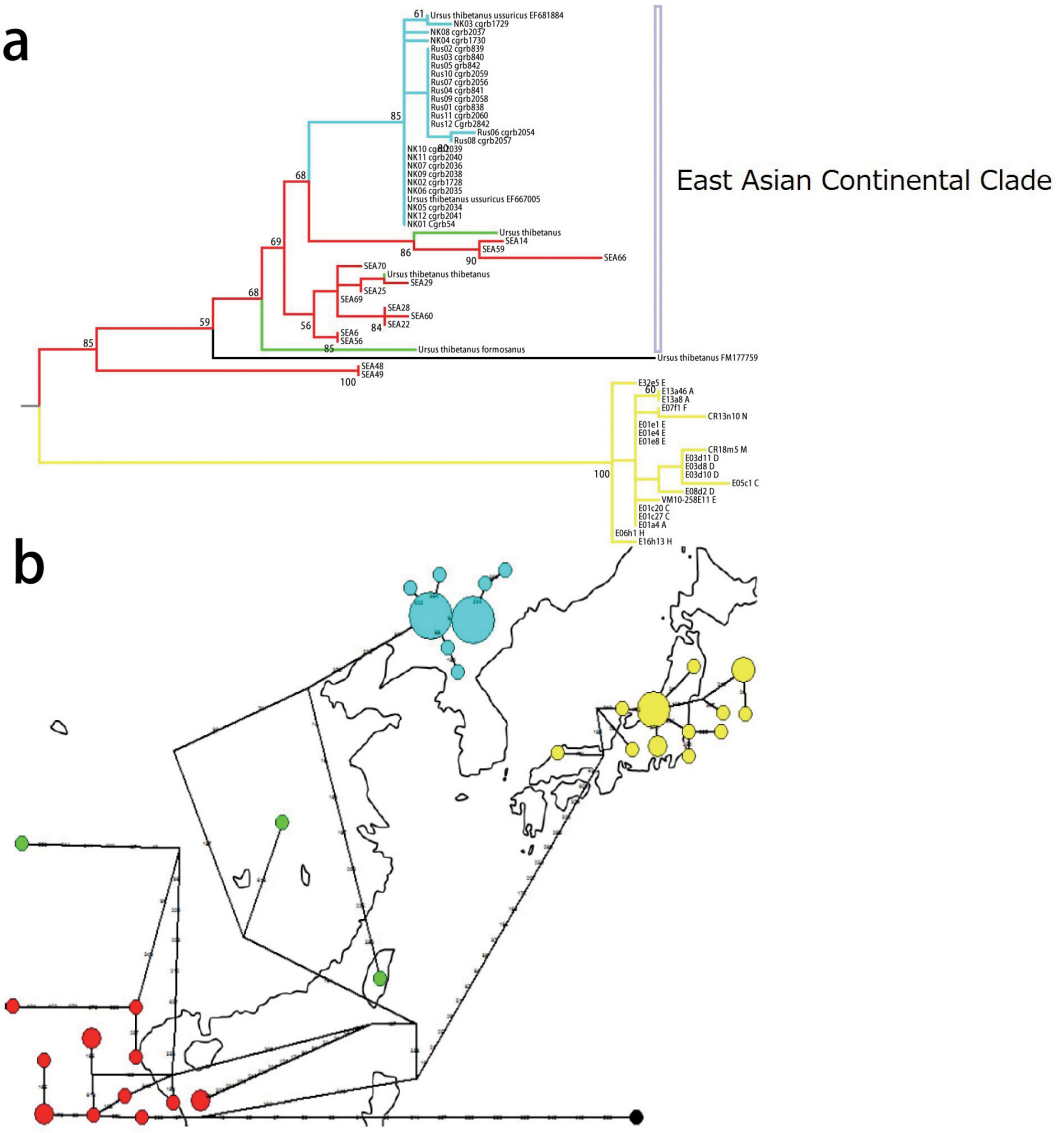
Phylogenetic relationships among Asian black bears. (a) The ML tree as inferred from the D-loop sequence. Nodal numbers indicate bootstrap probabilities (rapid bootstrap method with 1000 replications). The binary backbone structure was given a priori on the basis of the mitochondrial genome data (the phylogenetic relationships on the basis of the mitochondrial genome data are shown in Fig 2). Branch lengths are proportional to the number of nucleotide substitutions. (b) The median joining network. The circles indicate individual haplotypes, and their sizes are proportional to the frequency of the haplotypes. The short thick bars on the branches indicate unobserved ancestral haplotypes. In both (a) and (b), individuals from the Japanese population, the southern continental population (including Taiwan) and the northern continental population are colored yellow, red and light blue, respectively (branches are colored in (a), and haplotypes in (b)). Since the geographical information of FM177759 is unknown, it is colored black.

Subsequently, a population represented by the individual FM177759 [46] branched off. The geographical origin of this individual is unknown. Black bears in the East Asian continent (China, Korea, Russia) then diversified (the “East Asian continental clade,” which is constituted from part of the “South continental population” and the entire “North continental population”; Figs 2, 3 and 4). This species is also distributed in Taiwan (*U*. *thibetanus formosanus*). The black bear from Taiwan is highly nested within the South continental population, but because only one individual was involved in our analysis, our inclusion of the Taiwanese local population as part of the south continental population is tentative. The Korean sub-species (*U*. *thibetanus ussuricus*) is also highly nested in this East Asian continental clade, and can be recognized as the most recently evolved group. Concerning the phylogenetic relationships among black bears in the East Asian continent, there is an inconsistency between this study and Choi et al. [55], even though the regions used to establish the nucleotide sequence data sets in the two studies are almost identical. In our tree, the subspecies *U*. *t*. *formosanus* was placed at the basal position of the East Asian continental clade; the Chinese continental sub-species (*U*. *t*. *thibetanus*, *U*. *t*. *mupiensis*) successively diverged, and finally the Korean subspecies (*U*. *t*. *ussuricus*) was separated from the former subspecies. In Choi et al.’s tree [55], in contrast, the subspecies *U*. *t*. *mupiensis* diverged first and then the Korean subspecies (*U*. *t*. *ussuricus*) evolved; the other Chinese continental subspecies then diversified. The Taiwanese subspecies (*U*. *t*. *formosanus*) was recognized as the final split. These inconsistencies are probably due to a rooting problem of the East Asian continental clade. Indeed, except for the position of the root, our two tree toplogies are identical (if limited to the East Asian continental clade). In contrast to Choi et al. [55], who used other species (e.g., the American black bear and the brown bear) as out-groups of the East Asian continental clade, the Japanese subspecies and FM177759 (locality unknown) acted as an out-group in this study. Since an out-group that is closer to the in-group is preferable, the position of the root in this study seems to be more appropriate.

The genealogy inferred by the coalescent method also shows an identical branching pattern (Fig 3). Every fragment shows an identical genealogy. Although several nodes were recognized as multifurcation, due to the lack of mutation sites in some fragments, every node seen in Fig 2 and S3 Fig was supported by at least four fragments out of the six. Since different methods for the reconstruction of the geneaology (the phylogenetic method and the population genetic method) support the identical topology, we conclude that the genealogy presented in this study is robust and reliable.

Subsequently, a ML tree and a MJ network were also inferred based on the D-loop sequence data, and are shown in Fig 4A and 4B, respectively. This analysis includes the individuals from Southeast Asia. In contrast to Kim et al. [10], the basal position of the Japanese population among Asian black bears was still supported (85%). Since the binary backbone was applied for the tree inference, we were able to take advantage of the mitochondrial genome in our analysis. Therefore, the tree with the Japanese population at its base seems to be more plausible. Individuals from East Asia such as mainland China, Taiwan and Korea were highly nested and intermingled with individuals from Southeast Asia within the East Asian continental clade. From the criterion of parsimony, the ancestral distribution area of the continental population was probably Southeast Asia, and groups of individuals from that population migrated into China multiple times.

The coalescent times of the Asian black bear were estimated based on this tree topology using synonymous substitution sites of protein-coding genes, and the results are shown in Fig 2 (a magnified view of *U*. *thibetanus* is displayed). Since the effect of multiple substitutions at the synonymous sites was minor at the genus level (S4 Fig), the estimated divergence times within the genus *Ursus* were applied as calibration points for coalescent time estimations. The coalescent time between the Japanese population and the continental population was estimated to be 1.48±0.67 Ma. GENETREE analysis also yielded a similar result (1.20–1.52 Ma; average, 1.34 Ma). Previous studies addressing coalescent times within the species *U*. *thibetanus* yielded contradictory results [8,9]. Our estimate was slightly younger than Ohnishi et al.’s estimates (2.57–1.42 Ma) [8], and substantially older than Yasukochi et al.'s estimates (0.48–0.67 Ma) [9]. Ohnishi et al. [8] analyzed D-loop sequences to calculate the coalescent time between the Japanese and continental subspecies, using the *U*. *arctos/U*. *thibetanus* speciation as a calibration point. They assumed rapid speciation events within the genus *Ursus* at 3.5–2.0 Ma (Late Pliocene to Early Pleistocene) based on the fossil record [12]. However, Kurtén and Anderson [56] suggested that the species of this genus arose in the Early Pliocene (5.3–3.5 Ma) in the Old World. Therefore, calibrations that [8] used were too young; nevertheless, our time estimation is assuming the speciation within the genus *Ursus* at 6.3–3.9 Ma, but our estimate was younger than [8]’s estimates. Since the substitution rate in the D-loop sequence is very high, the effect of multiple substitutions becomes a serious problem in time estimation at the level of different species (S4 Fig). Accordingly, given that the *U*. *arctos*/*U*. *thibetanus* separation was used as a calibration point, coalescent times among the subspecies of *U*. *thibetanus* based on D-loop sequences should have resulted in a gross overestimation (i.e., older estimates) as was the case in Ohnishi et al.'s analysis [8]. This is why our estimate is younger than [8]’s estimates even though we used a much older time as a calibration point.

Yasukochi et al. [9] estimated coalescent times among *U*. *thibetanus* subspecies assuming a divergence time between *U*. *spelaeus* (cave bear) and *U*. *arctos* at 1.2 Ma. This date was estimated by Loreille et al. [11] based on D-loop sequences. However, they indirectly applied the evolutionary rate of homologous genes in human. The evolutionary rate among different orders of mammals is sometimes extremely different, and the substitution rate of mitochondrial genomes in Primates is high among mammals. Compared with Primates, the mitochondrial genomes of Carnivora evolve more slowly [57]. If the evolutionary rate of human homologous genes is applied, divergence times within the genus *Ursus* will be underestimated. In our analysis, the speciation of *U*. *spelaeus* and *U*. *arctos* was 2.6 Ma (95%CI: 3.39–1.96 Ma), which was directly estimated in the framework of whole carnivoran evolution using reliable fossil records. It is about 2.2 times older than [11]’s estimates. Since [9]’s estimation was based on *cytochrome b* sequences, in which substitutions are not saturated within the genus *Ursus*, if they had applied 2.6 Ma for the *U*. *spelaeus*/*U*. *arctos* speciation, their estimates would have been very close to ours.

Moving to the paleontological evidence, the fossil record of the Asian black bear in the Early Pleistocene is known only in Southeast Asia, but the geographical distribution of *U*. *thibetanus* became much wider in the Middle Pleistocene [58]. The tMRCA (time of the Most Recent Common Ancestor) of the continental population was estimated to be 0.98 Ma in this study. The most basal lineage in the continental population was the individual whose mitochondrial genome was sequenced by Krause et al. [46] (FM177759). Although geographical information about the individual is unknown, the split of this individual from other subspecies was considerably earlier. The coalescent times among continental subspecies in East Asian regions such as China, Korea, and Russia (East Asian continental clade) were estimated to be mainly between 0.21–0.52 Ma (Middle Pleistocene), which indicates that within this time period rapid sub-speciation occurred (the mitochondrial genome of the Asiatic black bear sequenced by Yu et al. [43] (NC009971) was from China; Dr. Li Yu, personal communication). This is consistent with the paleogeographical distribution of this species as shown by [58]. In the Middle Pleistocene, the distribution area of *U*. *thibetanus* became considerably more extensive than it is today, and fossil records have been reported from Southern Siberia, South and North Europe, and the middle Urals [2]. Successive splits of multiple mitochondrial lineages in this age seem to be correlated with the geographical expansion of this species.

### 3.3 tMRCA and population size of the Asian black bear

**Fig 5:**
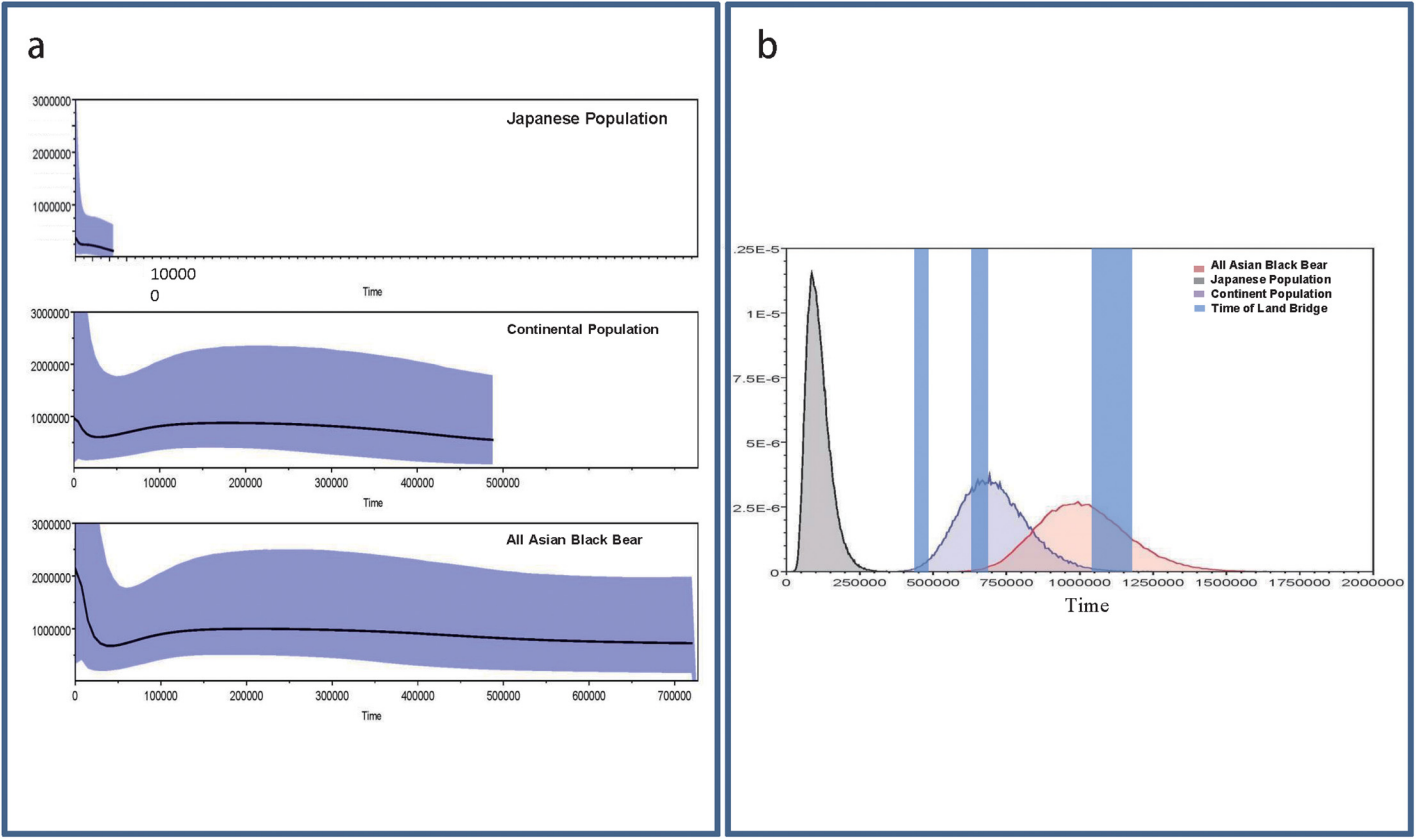
Dynamics of the population sizes and tMRCAs of all Asian black bears, the Japanese population and the continental population. (a) Dynamics of the population sizes estimated by Bayesian Skyline Plot analysis. The y-axes indicates the effective population size × generation intervals, and the x-axes indicate the time in years before present. (b) Posterior distributions of the tMRCAs. The times of formation of land bridges before the oldest record of the Japanese black bear (337–330 Kilo annum) are indicated by shaded bars, following Dobson and Kawamura [60] and Rohling et al. [63] with recalibrations by Lisiecki and Raymo [67]. The shading around the lines indicates 95% confidence interval of effective population size for each time point.

The tMRCAs and the dynamics of population sizes of all Asian black bears, namely the continental populations and the Japanese population, were estimated on the basis of mitochondrial D-loop data using the BEAST program, and the results are shown in Fig 5. As mentioned above, the Japanese population was divided into three sub-populations following [8]: Western sub-population, Eastern sub-population and Southern sub-population. The tMRCAs and the dynamics of population sizes of the three Japanese sub-populations were also estimated (S5 Fig). The y-axes of Fig 5 and S5 Fig are equal to Nef × t (the effective female population size times the generation length in years). Female Japanese black bears attain physiological puberty at 4 years [1]. Since the Nef × t of Japanese black bears was estimated to be about 370,000, the effective population size of the current Japanese black bear population is 185,000, assuming equal numbers of females and males. Although the actual size of the current Japanese black bear population is unknown, it is roughly estimated to be 13,000~21,000 [59]. However, considering 1,500 to 2,500 Japanese black bears are killed every year [1], the estimate of 13,000~21,000 individuals should be considered as a minimal estimation.

The tMRCA of all Asian black bears was estimated to be 1.00 Ma (95% CI: 1.32–0.73Ma). This is younger than the coalescent time of Asian black bears inferred from mitochondrial genome data (1.48±0.67 Ma), probably due to an underestimation of the numbers of multiple substitutions (especially transitions) of D-loop sequences in deep branches (data not shown). The tMRCAs of continental populations were estimated to be 0.70 Ma (95%CI: 0.94–0.49Ma) and that of the Japanese population was estimated to be 0.10 Ma (95%CI: 0.19–0.04 Ma). Notably, the tMRCA of the Japanese population is only 1/10 of the tMRCAs of all Asian black bears. In addition, no fossil evidence of the Japanese population is known prior to the Late Pleistocene [1,58,60] (although unpublished fossil data from N. Kohno are discussed below). It is thus likely that although the Japanese population is placed as the basal lineage among all other populations of *U*. *thibetanus* (Figs 2–4), the following two hypotheses are possible. (1) The animals dispersed into the Japanese Archipelago relatively recently (i.e., during the Late Pleistocene), or (2) they migrated earlier (the Early to Middle Pleistocene) and experienced recent population growth.

Although we cannot exclude the possibility of a recent migration event (Late Pleistocene) for the Japanese black bear, an old migration and recent population expansion may be a more plausible hypothesis. A possible explanation of the discrepancy between their distinctive old split from the continental population in the Early Pleistocene and their potential recent tMRCA of the population in the Japanese main islands (i.e., Honshu, Shikoku and Kyushu) after the late Middle Pleistocene may be the re-dispersal of a partially restricted population of Japanese black bears into the newly emerged ecological empty space caused by the reduction and extinction of the brown bear (*U*. *arctos*) population; an exotic larger species in the Pleistocene of Honshu exploited the same resources in a similar manner. According to [61, 62], the Pleistocene mammalian fauna of the Japanese main islands included the brown bear until the Late Pleistocene. Their possible reduction in the late Middle Pleistocene and extinction in the Late Pleistocene might have been the result of fragmentation of forests in Honshu, and might have resulted in an abrupt emergence of an empty niche for the Japanese black bear after the late Middle Pleistocene in the Japanese main islands. The population sizes of all Asian black bears (comprising continental and Japanese populations) increased slightly after the last glacial period. The Japanese population expanded considerably throughout the late Middle to the Late Pleistocene (Fig 5, Table 1). This scenario also supports “the old migration and recent population growth" hypothesis.

**Table 1:** Summary Statistics for the demography.

population	N	Θπ	Θw	Tajima'sD*
All	64	0.02423	0.02354	0.037314
continent	44	0.02019	0.01981	-1.11146
Southern Continent	17	0.0202	0.01972	-0.63669
Northern Continent	26	0.00296	0.00296	-1.19211
Japan	760	0.00979	0.00957	-1.33351
Western Japan	130	0.00386	0.00386	0.77907
Eastern Japan	617	0.0068	0.00657	-1.39115
Southern Japan	13	0.0031	0.0031	0.71469

N: numbers of the samples

Θπ: Theta based on the nucleotide diversity

Θw: Waterson's theta

*All of them were not significant in this study (p-values> 0.1)

Although paleontological evidence does not show the bears' precise migratory history [1,58, 60], phylogeographically there are at least three possible migration routes from the Asian continent to the Japanese archipelago, and particularly Honshu. The first is the Siberia-Sakhalin-Hokkaido route via the Mamiya Strait (between Siberia and Sakhalin), the Soya Strait (between Sakhalin and Hokkaido), and the Tsugaru Strait (between Hokkaido and Honshu). The second possibility is the Ryukyu archipelago route, and the third is the Korean Peninsula to Honshu+Kyushu route via the Korean and Tsushima Straits. Since the Japanese population of Asian black bears is distributed only in the Japanese main islands, and no extant population or fossils have ever been found in Hokkaido or Ryukyu, the first and second possible routes are unlikely. It is also known that the Tsugaru and Tsushima Straits did not form land bridges during the Last Glacial Maximum (LGM) period in the Late Pleistocene [63]. Therefore, the Korean Peninsula–Japanese main islands route also seems unlikely for the migration of Japanese black bears during the LGM. This means that the Japanese black bear appeared in the Japanese Islands before their population expansion during the late Middle and Late Pleistocene. Taruno [64] and Takahashi [65] suggested the possibility that a transient land bridge formed between the Asian continent and Japanese Archipelago during the Early Pleistocene (1.2–1.0 Ma [66, 67]) and the Middle Pleistocene (0.678–0.621 Ma and 0.478–0.424 Ma [66, 67]). The timing of the occurrence of this land bridge in the Early Pleistocene is consistent with the divergence time between the Japanese and continental subspecies (the tMRCA of all Asian black bears).

**Fig 6:**
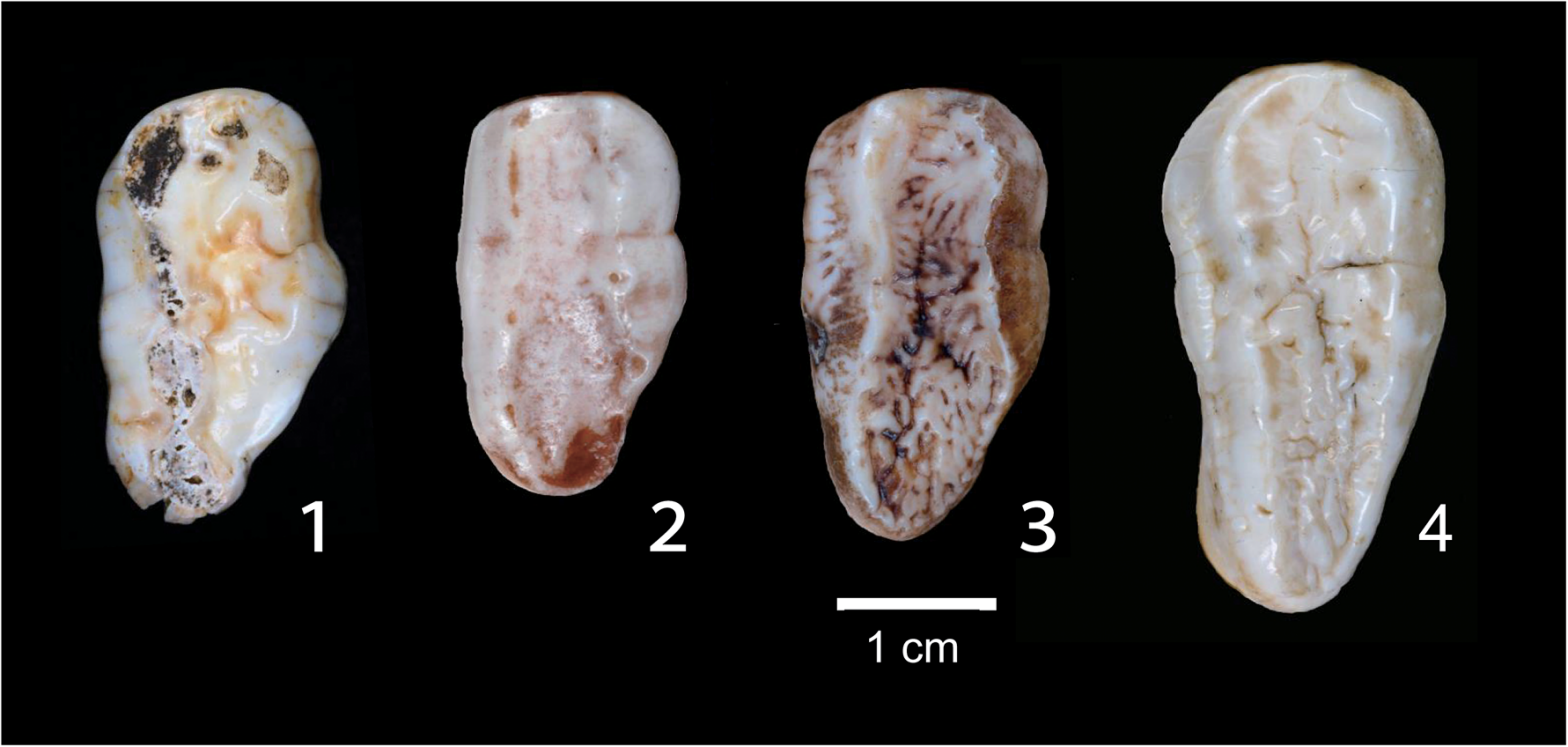
Middle Pleistocene black bear from Japan with a comparison of the left upper second molars (M2) among bears. 1, Middle Pleistocene (ca. 337–330 Kilo annum, MIS 9 [68]) black bear, *Ursus thibetanus* subsp. indet., from Aomori Prefecture, northern Japan (NMNS-PV 22666). 2, Extant Japanese black bear, *Ursus thibetanus japonicus*, from Nagano Prefecture, central Japan (NMNS-PO 52). 3, Extant Tibetan black bear, *Ursus thibetanus thibetanus*, from Thailand (NMNS-PO 207). 4, Extant Hokkaido brown bear, *Ursus arctos yesoensis*, from Hokkaido, northern Japan (NMNS-PO 208). The black bears share a combination of M2 characters such as a relatively large metacone (as large as the paracone), a distinct constriction between the paracone and the metacone, and a less developed posterior talon. In contrast, the brown bear has a smaller metacone relative to the paracone, a less distinct constriction between the two cusps, and a well developed posterior talon in M2. These comparisons suggest that the tooth of Middle Pleistocene Japanese black bear is closer in size and shape to the teeth of continental black bears than it is to extant Japanese black bears, but it is far from the brown bears.

Without fossil evidence of the existence of the Japanese black bear in the Japanese Archipelago in the Early and Middle Pleistocene, it was difficult to claim that the ancient population of the Japanese subspecies had lived there before the Late Pleistocene and that they migrated from the continent during the late Early to the Middle Pleistocene, by passing such a land bridge. However, the recent discovery of a Japanese black bear fossil from the late Middle Pleistocene (MIS 9 or about 337 Kilo annum [68]) in Aomori Prefecture (Fig 6) demonstrated that the Asian black bear lived at least in Honshu by the late Middle Pleistocene before the population expansion of the Japanese black bear in the Late Pleistocene in the Japanese Islands. In this regard, the existence of the brown bear [1,58,60] in Honshu might have confined the Japanese black bear to restricted areas (e.g., mountains) in Honshu during the Middle Pleistocene. As mentioned above, the Japanese population depends more on vegetation than meat compared with the continental population [1]. It is possible that the high dietary dependence on vegetation in the Japanese population was an adaptation to avoid competition with the brown bear. After the reduction and extinction of the brown bear, which accords with the reduction and/or fragmentation of forests and resultant extinction of large herbivores, the ecological niche of brown bear in Honshu was evidently replaced by the Japanese black bear, whose population subsequently expanded to the east and south in the Japanese main islands.

To date, Japanese-type mitochondrial haplotypes have not been reported from the continent (e.g., [10]). After the ancestral population of the Japanese black bear migrated from the continent to the Japanese Archipelago, probably via the Korean Peninsula in the Early to the Middle Pleistocene glacial period, they became extinct or were drastically reduced on the continent, especially in Northern Asia. The Japanese Islands may therefore have been a refugium for the ancestral Asian black bear. Any of the ancestral population remaining in North Asia may have been replaced by the newly evolved subspecies *ussuricus* in the Middle Pleistocene. However, since genetic data from the continental population are very limited (only 44 individuals from four countries), more extensive analysis of the continental population, focusing both on geographical variation and on numbers of individuals, will shed further light on the enigmatic history of the Asian black bear.

## Supporting Information

S1 Fig Dynamics of the population of the protein coding genes (about 10 kbp) and D-loop (about 500 bp) on the basis of real and simulated nucleotide sequence data. The dynamics of the population sizes estimated by Bayesian Skyline Plot analysis are shown. Vertical axes indicate the effective population size × generation intervals; horizontal axes indicate time in years before present. The shading around the lines indicates 95% confidence interval of effective population size of each time point.
(PDF)

S2 Fig The ML tree of 71 Carnivora and 1 Pholidota as inferred from the complete mitochondrial protein genes. Numbers at nodes indicate bootstrap probability (BP) in % (1000 replications). Branch lengths are proportional to the number of nucleotide substitutions.
(PDF)

S3 Fig The ML tree of eight Asian black bears as inferred from the complete mitochondrial protein genes and rRNAs. Numbers at nodes indicate BP in % (1000 replications). Branch lengths are proportional to the number of nucleotide substitutions.
(PDF)

S4 Fig Saturation plot analysis of the synonymous and non-synonymous sites of mitochondrial protein coding genes and D-loop sequence within Ursus thibetanus and among species of the genus Ursus. (PDF)

S5 Fig Dynamics of the population sizes and tMRCAs of the Japanese population, and its three constituent subpopulations (a) Dynamics of the population sizes estimated by Bayesian Skyline Plot analysis. Vertical axis indicates the effective population size × generation intervals. Horizontal axis indicates the time in years before present. (b) Posterior distributions of the tMRCAs.
(PDF)

S1 Table Species names and GenBank accession numbers of the complete mitochondrial genome data used in this study. (XLSX)

S1 Text Reliability of the Demographic Analysis Based on the D-loop Sequences. (DOCX)
