# Supplementary material for: Phylogeographic and Demographic Analysis of the Asian Black Bear (Ursus thibetanus) Based on Mitochondrial DNA
Source: PLoS One. 2015 Sep 25;10(9):e0136398. doi: 10.1371/journal.pone.0136398 (PMC4583410; doi:10.1371/journal.pone.0136398)

Simulated data (birth rate:2, death rate:2, sampling fraction: 0.1)

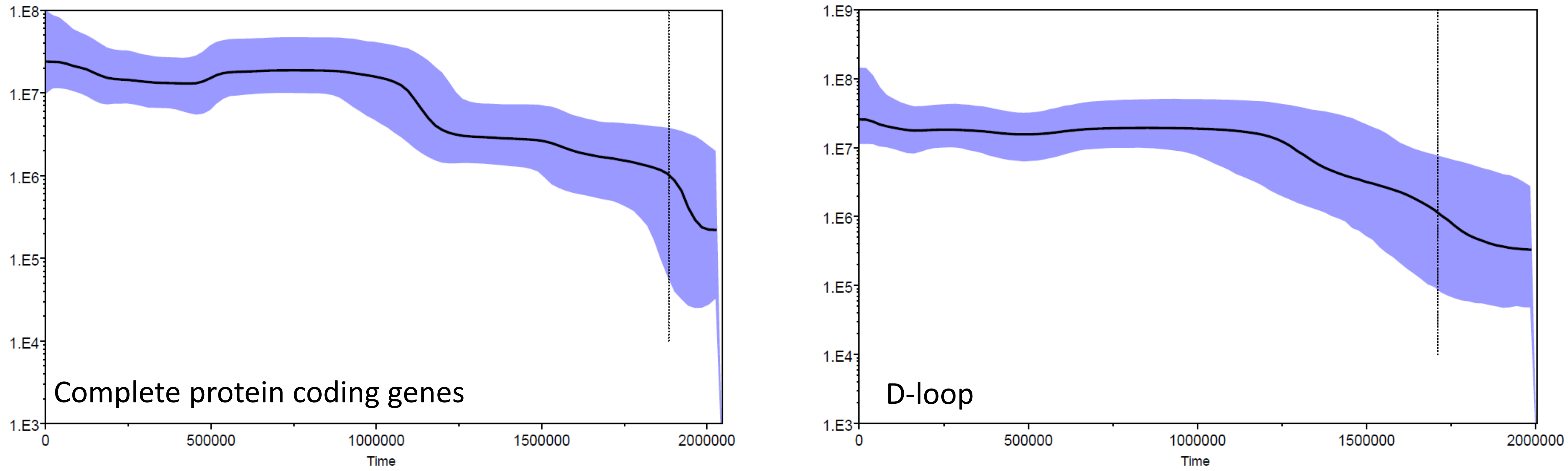

Simulated data (birth rate:2, death rate:2, sampling fraction: 0.9)

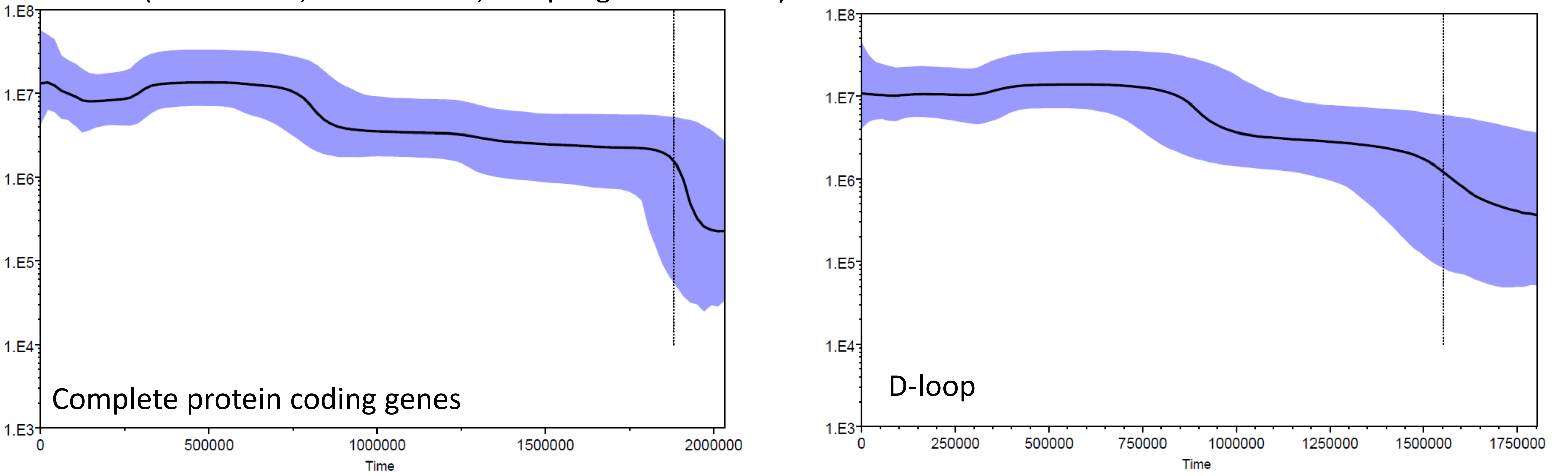

Simulated data (birth rate:10, death rate:5, sampling fraction: 0.01)

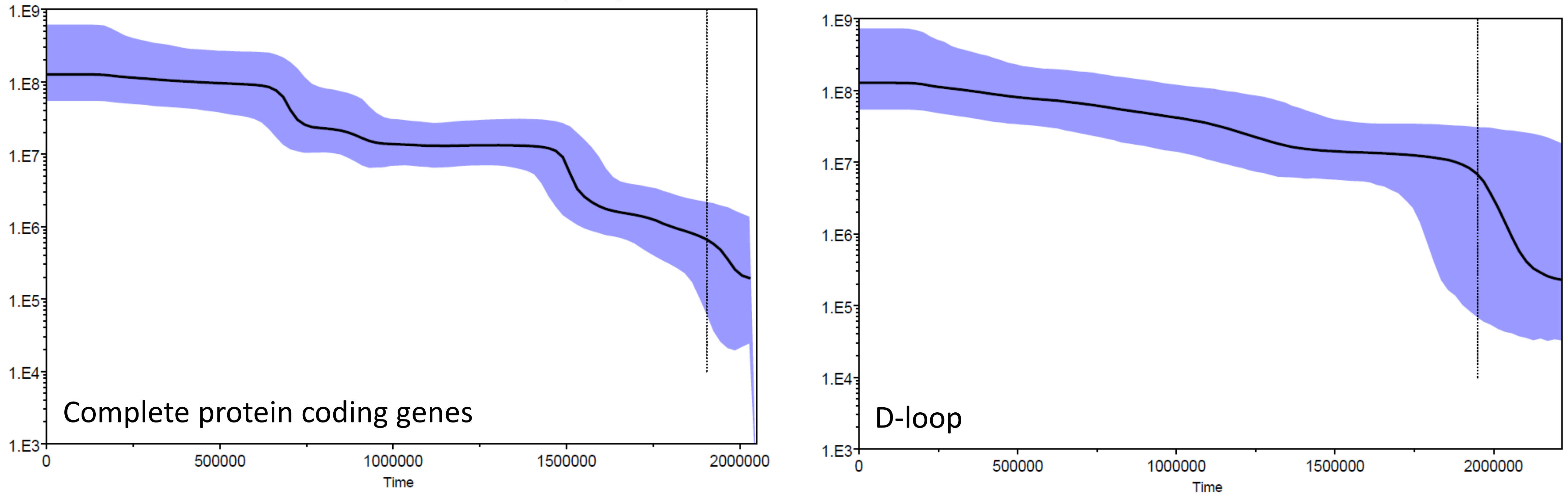

Simulated data (birth rate:10, death rate:5, sampling fraction: 0.999)

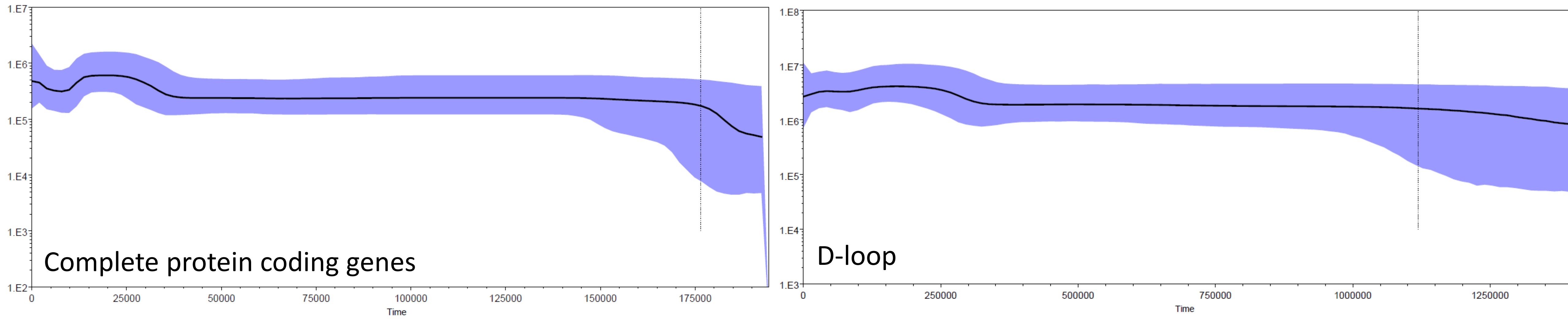

Real data (Keis et al. (2013)'s data of brown bears in north-west Eurasia)

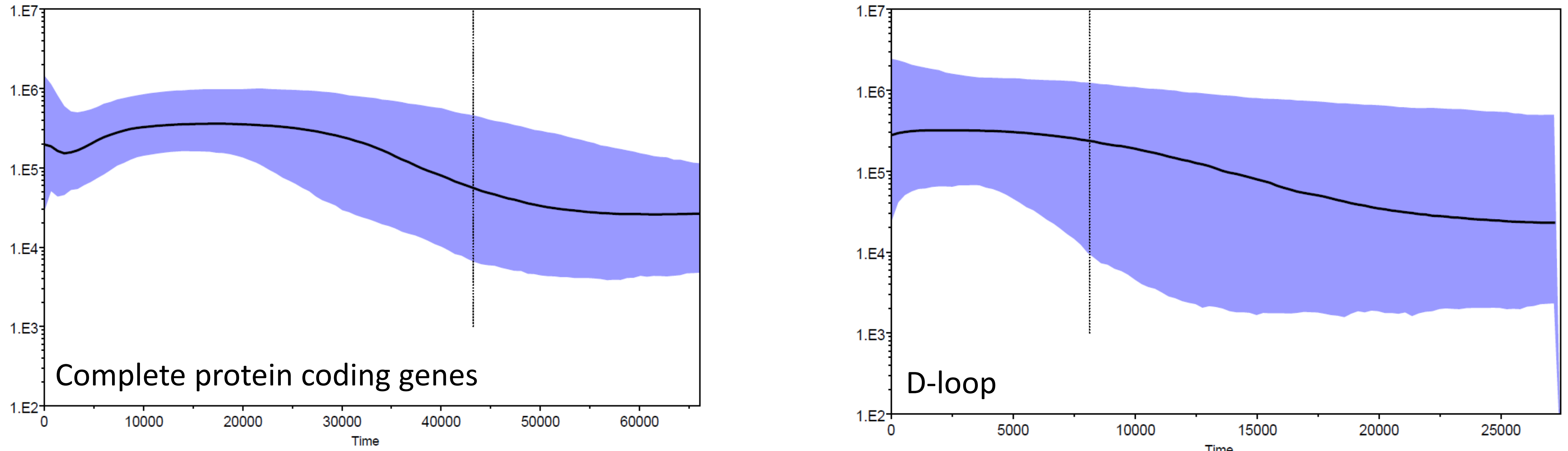

Supplement: S1 Fig — The dynamics of the population sizes estimated by Bayesian Skyline Plot analysis are shown. Vertical axes indicate the effective population size × generation intervals; horizontal axes indicate time in years before present. The shading around the lines indicates 95% confidence interval of effective population size of each time point. (PDF) [file pone.0136398.s001.pdf]
