# Supplementary figures and images for: Phylogeographic and Demographic Analysis of the Asian Black Bear (Ursus thibetanus) Based on Mitochondrial DNA
Source: PLoS One. 2015 Sep 25;10(9):e0136398. doi: 10.1371/journal.pone.0136398 (PMC4583410; doi:10.1371/journal.pone.0136398)

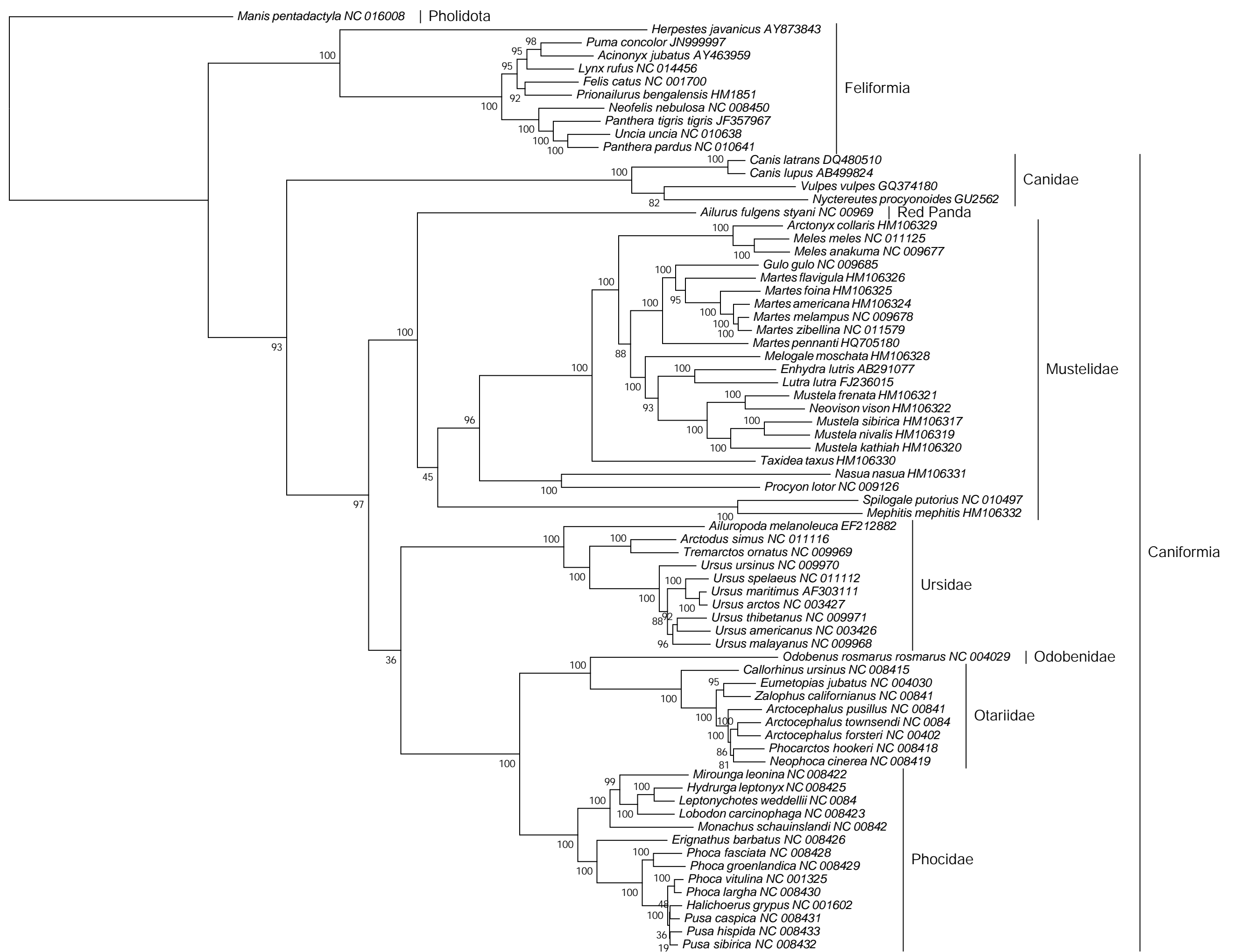

Supplement: S2 Fig — Numbers at nodes indicate bootstrap probability (BP) in % (1000 replications). Branch lengths are proportional to the number of nucleotide substitutions. (PDF) [file pone.0136398.s002.pdf]

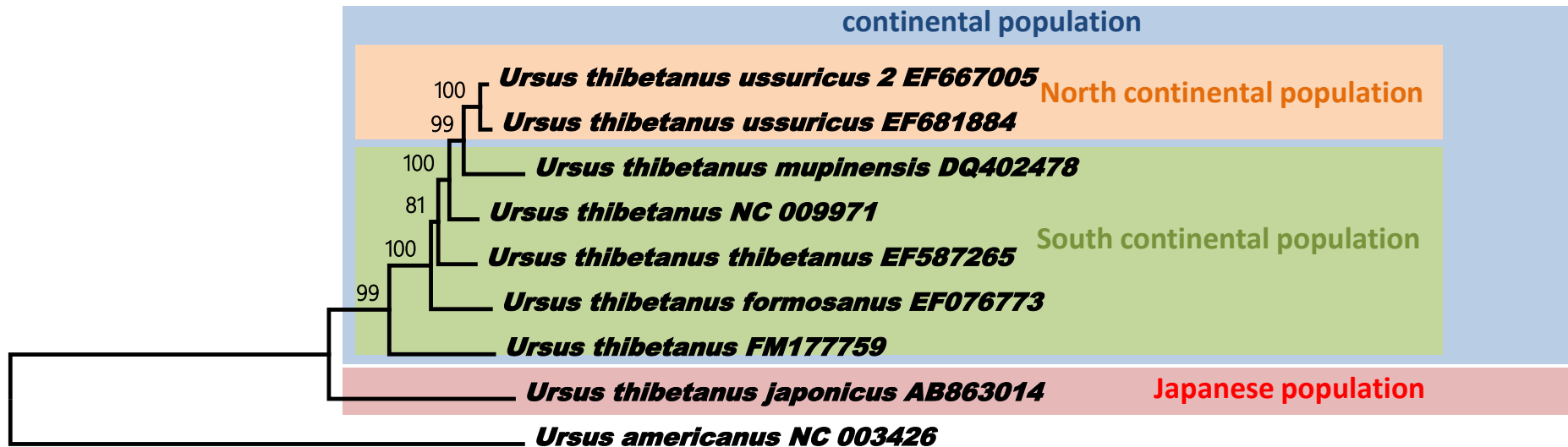

0.02

Supplement: S3 Fig — Numbers at nodes indicate BP in % (1000 replications). Branch lengths are proportional to the number of nucleotide substitutions. (PDF) [file pone.0136398.s003.pdf]

# P-distance of D-loop and uncorrected dS (mt genome and cyt b)

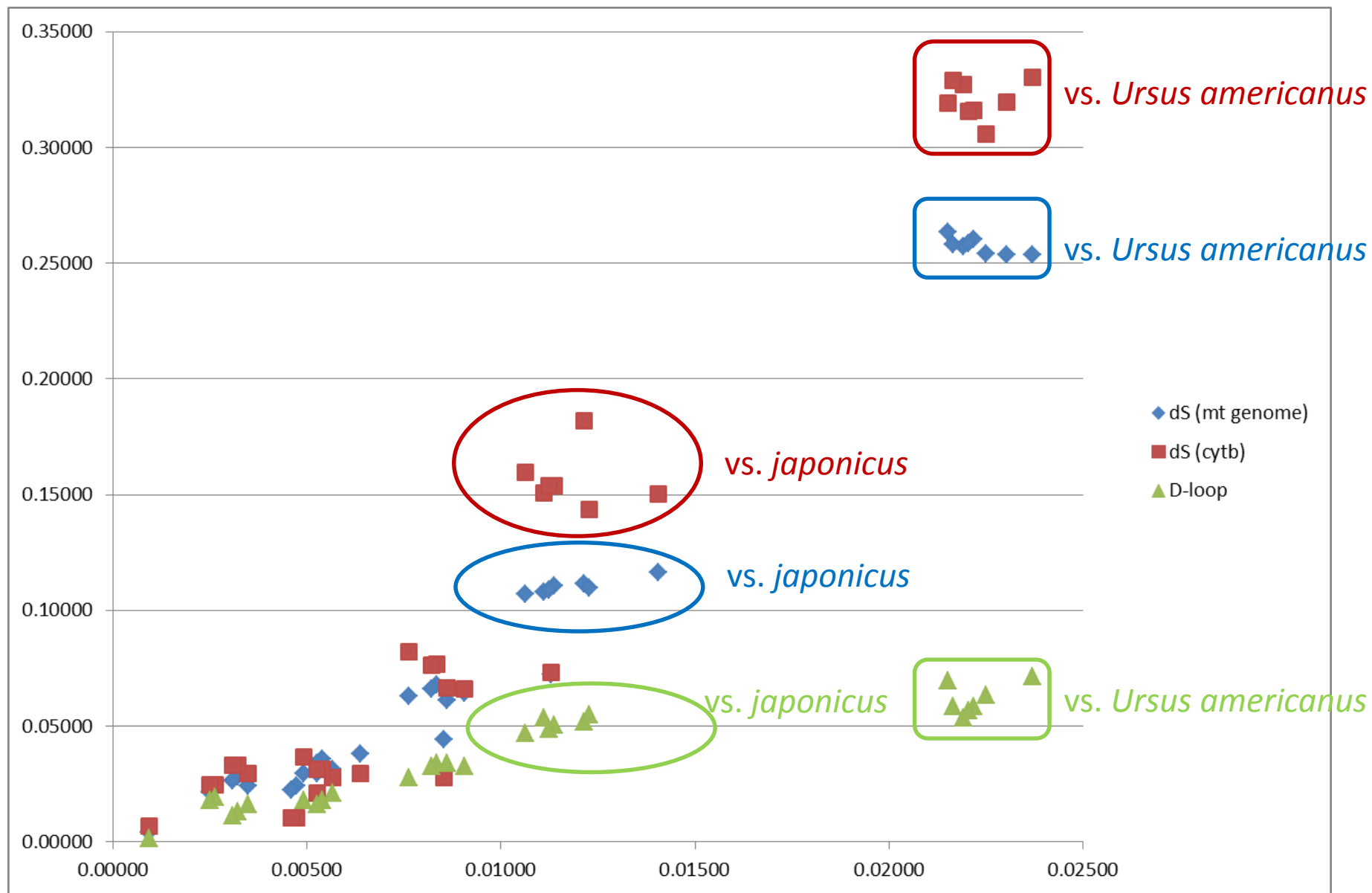

Uncorrected dN of mt genome

Supplement: S4 Fig — (PDF) [file pone.0136398.s004.pdf]

**a**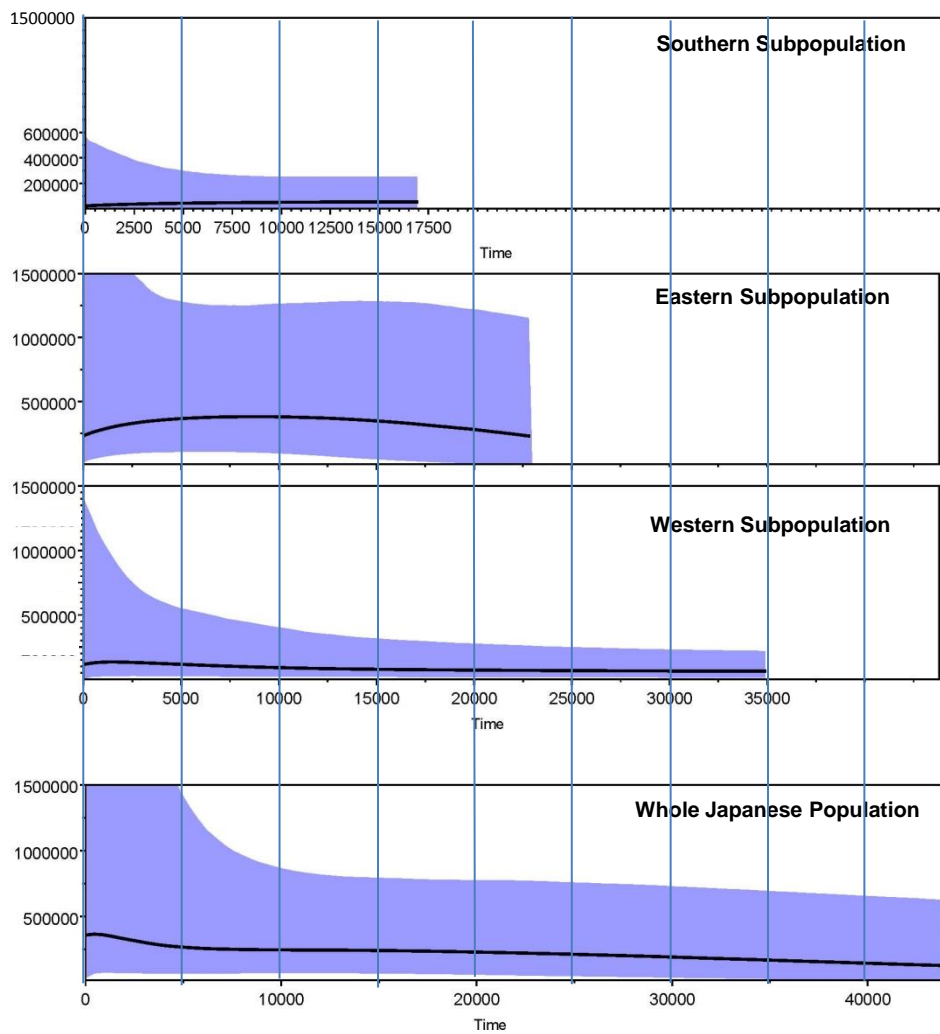**b**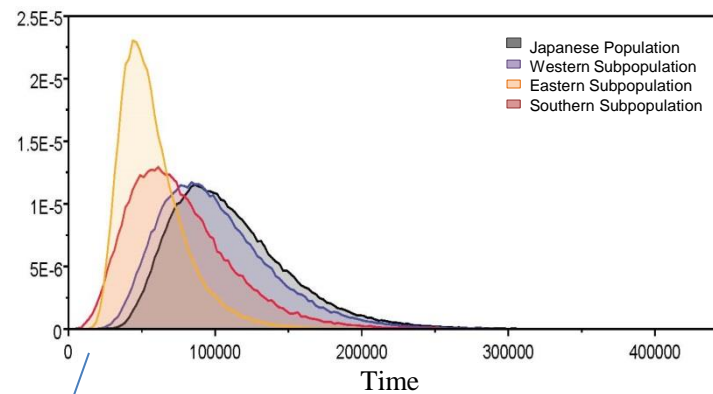

Supplement: S5 Fig — Vertical axis indicates the effective population size × generation intervals. Horizontal axis indicates the time in years before present. (b) Posterior distributions of the tMRCAs. (PDF) [file pone.0136398.s005.pdf]
