# Supplementary material for: Phylogeographic and Demographic Analysis of the Asian Black Bear (Ursus thibetanus) Based on Mitochondrial DNA
Source: PLoS One. 2015 Sep 25;10(9):e0136398. doi: 10.1371/journal.pone.0136398 (PMC4583410; doi:10.1371/journal.pone.0136398)
Supplement: S1 Text — (DOCX) [file pone.0136398.s007.docx]

**Supplemental Text**

**Reliability of Demographic Analysis Based on D-loop Sequences**

Since the D-loop sequences are short (623 bp for the partial D-loop sequences used in this study), and have an issue regarding homoplasy caused by multiple substitutions at the same sites, one may doubt the reliability of the demographic analysis based on these D-loop sequences. With the aim of addressing this issue, the reliability of the analyses of D-loop sequences was evaluated using real and simulated sequence data. For the analysis of the real data, the complete mitochondrial genomes of brown bears in north-western Eurasia sequenced by Keis et al. [1] were used. The D-loop sequences, whose positions are homologous with that of the D-loop sequence in our study, as well as the codon sequences of the 12 protein coding regions on the H strand were analyzed. The simulated data were generated by the Evolver program in PAML [2]. A total of 3333 codon sites (9999 nucleotide sites) were generated as a CDS, and 500 nucleotide sites were generated as a D-loop. We assumed that the evolutionary rate of the D-loop is 30 times faster than that of the CDS. Bayesian Skyline Plot analysis was then carried out using the BEAST program. The HKY85+Γ model was used and the strict clock model was assumed for this analysis. The proportion of the tree topologies were determined by the “birth rate”, “death rate” and “sample fraction”. Although these parameters are originally for the inter-species concept, we used them as an approximation of the parameters for the demography.

The results are shown in S1 Fig. Both the real and the simulated sequence data indicate that although the resolution of the demography based on the complete protein coding genes is generally better, the demography on the basis of the D-loop sequences and the complete protein coding genes shows a fundamentally similar tendency. On this foundation, as far as the rough tendency is taken, the demography on the basis of the D-loop sequences is also reliable.

[1] Keis M, Remm J, Ho SYW, Davison J, Tammeleht E, Tumanov IL, Saveljev AP, Mannil P, Kojola I, Abramov AV, Margus T, Saarma U. (2013) Complete mitochondrial genomes and a novel spatial genetic method reveal cryptic phylogeographical structure and migration patterns among brown bears in north-western Eurasia. J. Biogeogr. 40: 915–927.

[2] Yang Z (2007) PAML 4: phylogenetic analysis by maximum likelihood. Mol Biol Evol 24: 1586-1591.
